# Preparation of Vitexin Nanoparticles by Combining the Antisolvent Precipitation and High Pressure Homogenization Approaches Followed by Lyophilization for Dissolution Rate Enhancement

**DOI:** 10.3390/molecules22112038

**Published:** 2017-11-22

**Authors:** Chengbo Gu, Ziwei Liu, Xiaohan Yuan, Wang Li, Yuangang Zu, Yujie Fu

**Affiliations:** 1 Key Laboratory of Forest Plant Ecology, Ministry of Education, Northeast Forestry University, Harbin 150040, China; ziwei17liu@163.com (Z.L.); wanglisouthernsea@126.com (W.L.); zuyuangang@163.com (Y.Z.); 2 Life Science and Biotechnique Research Center, Northeast Agricultural University, Harbin 150030, China; yuanxiaohana@163.com

**Keywords:** nanotechnology, nanoparticles, vitexin, dissolution rate

## Abstract

Vitexin, a natural flavonoid found in many medicinal plants, is well known for its rich pharmacological activities. However, the poor water solubility of vitexin has limited its therapeutic application. The aim of this study was to prepare the nanoparticles of vitexin by combining the antisolvent precipitation (ASP) and high pressure homogenization (HPH) approaches followed by lyophilization for improving the dissolution rate of this poorly water-soluble drug. The effects of main factors influencing the mean particle size (MPS) of vitexin were investigated and optimized. Under optimum conditions, vitexin nanosuspensions with an MPS of 80.5 nm were obtained and then lyophilized to form nanoparticles. The obtained vitexin nanoparticles were further characterized by scanning electron microscopy (SEM), Fourier transform infrared spectroscopy (FTIR), mass spectrometry (MS), X-ray powder diffraction (XRPD), gas chromatography (GC) and dissolution testing. The results showed that the nanoparticles of vitexin were converted into an amorphous form, with its chemical structure unchanged. Additionally, the residual dimethyl sulfoxide (DMSO) is lower than the International Conference on Harmonization (ICH) limit for class 3 solvents. The dissolution rate of processed vitexin was significantly higher (5.58-fold) than that of raw drug. Overall, the combinative process we developed is an effective way to produce vitexin nanoparticles with markedly enhanced dissolution rate.

## 1. Introduction

**Figure 1:**
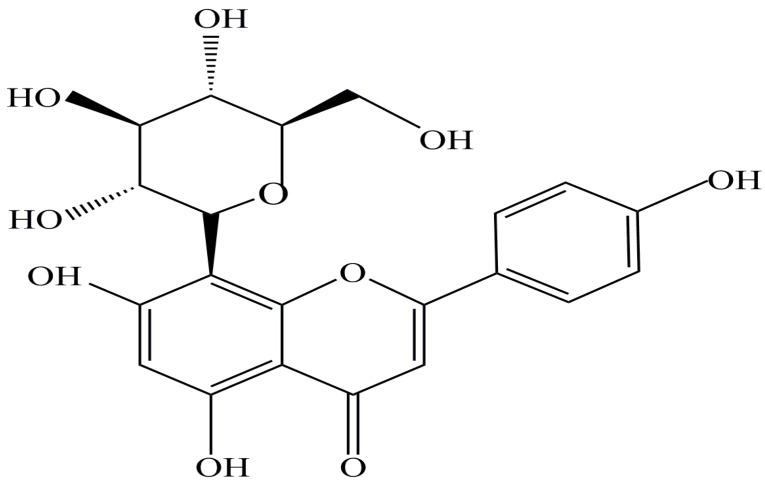
Chemical structural formula of vitexin.

Vitexin (apigenin-8-C-glucoside, as shown in Figure 1) is a naturally occurring flavone glucoside, which was found in many medicinal plant species such as *Crataegus pinnatifida* [1], *Cajanus cajan* [2], *Livistona chinensis* [3], *Mimosa pudica* [4], *Phyllostachys edulis* [5], and *Trollius chinensis* [6]. Vitexin has received increasing attention due to its various pharmacological effects, such as antihypotensive, anti-inflammatory, anticancer, antispasmodic, antimicrobial, antioxidant, antithyroid and antiarteriosclerotic effects [7,8]. It is also used for heart disease prevention [9].

Despite having multiple medicinal benefits, the low aqueous solubility and poor bioavailability of vitexin limit its clinical application [9,10]. To improve the water solubility of vitexin, micronized vitexin particles was made using the supercritical antisolvent technology, but it has the limitations of low yield and high equipment cost during the preparation process [9].

Nanoparticle engineering is one of the recent strategies applied to solve the problems of poorly aqueous soluble compounds [11]. Standard methods employed for the preparation of nanoparticles can be basically classified into top-down and bottom-up techniques [12]. In top-down methods, nanoparticles are produced by breaking down the large particles to smaller particle sizes by mechanical attrition such as high pressure homogenization (HPH) and wet milling [13,14,15]. Although the top-down approaches are useful on the commercial front, these processes suffer from certain drawbacks like high energy costs, time-consuming and broad particle size distributions [16,17]. For the bottom-up approach, nanoparticles were produced by precipitation. The antisolvent precipitation (ASP) is attractive to most of the bottom-up methods. In this process, the drug is dissolved in a water-miscible organic solvent and then precipitated by addition of an antisolvent under rapid mixing [18,19,20]. One of the main challenges of this process is the crystal growth after the precipitation [21].

In order to improve the particle size reduction effectiveness, a combination of two techniques (bottom-up and top-down) has also been employed, such as Nanoedge (a combination of precipitation and HPH) and Smart Crystal (H42 process: a combination of spray drying with HPH, and the H96 process: a combination of freeze drying and HPH) [22].

However, three combination technologies have been scarcely explored for preparation of nanoparticles. In the present study, we employed a combination of ASP and HPH approaches followed by lyophylization to prepare vitexin nanoparticles. Various factors affecting the mean particle size (MPS) of vitexin, including vitexin solution concentration, antisolvent to solvent volume ratio, stirring speed, precipitation temperature, homogenization pressure and cycle number, were investigated and optimized by an orthogonal array design (OAD) and single factor optimization design. The obtained vitexin nanoparticles were analyzed through scanning electron microscopy (SEM), Fourier transform infrared spectroscopy (FTIR), liquid chromatography-mass spectrometry (LC-MS), X-ray powder diffraction (XRPD), gas chromatography (GC), and dissolution testing. To the best of our knowledge, there is no study exploring the use of a combination method of ASP-HPH and lyophilization to produce vitexin nanoparticles.

## 2. Results and Discussion

### 2.1. Optimization of the ASP Process

The first step in the ASP process is to optimize the operating conditions to obtain a minimum MPS of micronized vitexin. In the present study, an orthogonal array design OAD9 (3^4^) was applied to evaluate the effects of the following factors on the MPS of vitexin particles: concentration of the vitexin solution (A), antisolvent to solvent volume ratio (B), stirring speed (C), and precipitation temperature (D). The assignment of the experiment and the collected data for MPS of micronized vitexin is in Table 1. The results in Table 1 indicated that the maximum MPS of vitexin particles was 1129.2 ± 95.25 nm, and the minimum was 368.4 ± 31.23 nm. It can be concluded from Table 1 that the influence to the MPS of micronized vitexin decreases in the order: D > A > B > C according to the *R*-values. In addition, the minimum MPS of vitexin particles was obtained by numerical analysis using the Design-Expert 8.0.5 software (Stat-Ease Inc., Minneapolis, MN, USA) as below: when vitexin solution concentration, antisolvent-solvent volume ratio, stirring speed, and reaction temperature were A1B2C3D1 (25 mg/mL, 15 times, 1500 rpm, and 4 °C), respectively. Through a validation test, smaller micronized vitexin was obtained with a MPS of 368.4 ± 31.23 nm.

The result of ANOVA analyses was shown in Table 2, the effect of the reaction temperature on the MPS of vitexin particles was significant (*p* < 0.05) among various experimental factors, and the concentration of vitexin solution, antisolvent to solvent volume ratio and stirring speed were not significant (*p* > 0.05).

### 2.2. Effect of Different Influential Factors on the MPS of Vitexin Particles in the ASP Process

**Figure 2:**
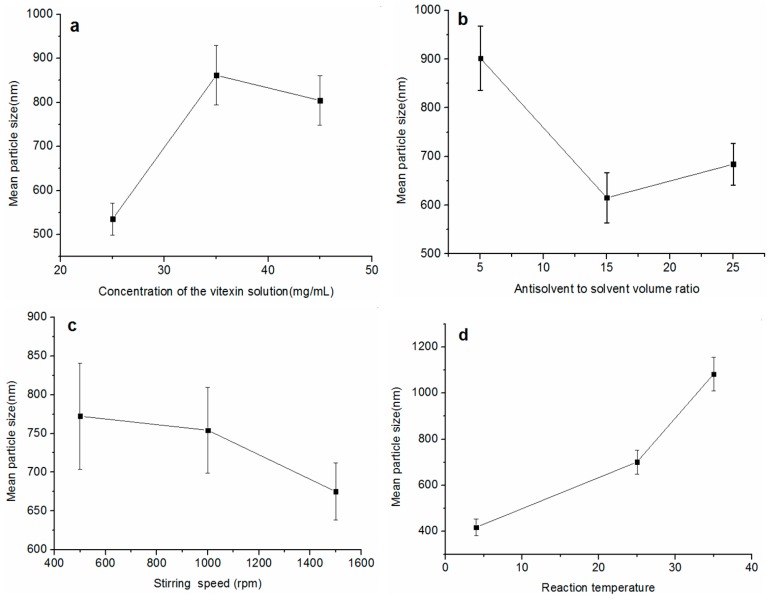
The effect of each factor on the MPS of vitexin particles. (**a**) concentration of the vitexin solution; (**b**) antisolvent to solvent volume ratio; (**c**) stirring speed; (**d**) reaction temperature.

The influences of various factors on the MPS of the vitexin particles are observed in Figure 2. The MPS of vitexin particles increased with vitexin solution concentration ranging from 25 to 35 mg/mL, but decreased when drug concentration increasing from 35 to 45 mg/mL (as shown in Figure 2a). The MPS of vitexin particles decreased with the antisolvent to solvent volume ratio increasing from 5 to 15 times, but increased when volume ratio varied from 15 to 25 times (as shown in Figure 2b). From Figure 2c, with the stirring speed increasing from 500 to 1500 rpm, the MPS of vitexin particles decreased significantly. Figure 2d showed that the reaction temperature had a large influence on particle size. As the reaction temperature changed from 4 to 35 °C, vitexin particle size increased significantly.

### 2.3. Optimization of HPH Process

**Figure 3:**
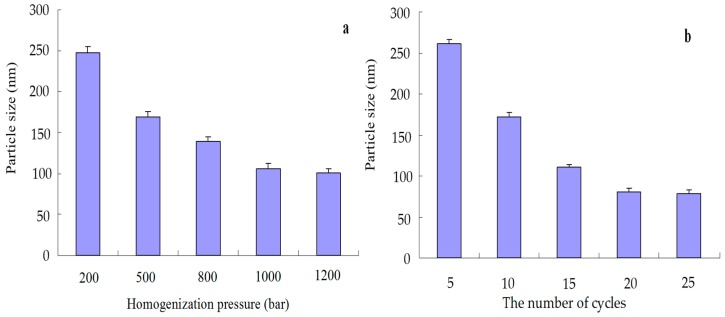
Effect of homogenization pressure (**a**) and homogenization cycles (**b**) on the particle size of vitexin nanosuspensions.

After ASP process is done, the parameters in the HPH process, such as homogenization pressure and homogenization cycles, were further optimized by single factor optimization design (shown in Figure 3). When keeping the numbers of homogenization cycle at 9, the operating conditions of homogenization pressures (200–1200 bar) were optimized, as shown in Figure 3a, increasing homogenization pressure from 200 to 1000 bar, the particle size fell. However, the higher pressure didn’t lead to significant improvement in particle size when the homogenization pressure exceeded 1000 bar. Thus, the optimal homogenization pressure was considered to be 1000 bar in the present experiment.

The number of homogenization cycles is another important factor determining the fineness of the product in the HPH process. Samples were withdrawn after 5, 10, 15, 20 and 25 homogenization cycles under 1000 bar (Figure 3b). Although more cycles lead to particle size reduction, from the lower energy consumption point of view, 20 homogenization cycles were good enough to reach a homogenous nanosuspension. Therefore, the optimized HPH condition was achieved by 1000 bar for 20 cycles. One important role of the HPH step is to anneal the initial precipitates obtained by the ASP process [23].

### 2.4. Characterization of Vitexin Nanoparticles

Vitexin nanosuspensions produced under the optimal operating conditions in ASP and HPH processes were transformed into nanoparticles by lyophilization using 5% mannitol as the cryoprotectant, and the vitexin nanoparticles were further analyzed in terms of morphology (SEM), particle size, XRPD, LC-MS, FTIR, GC, and dissolution behavior.

#### 2.4.1. SEM and Particle Size Analysis

**Figure 4:**
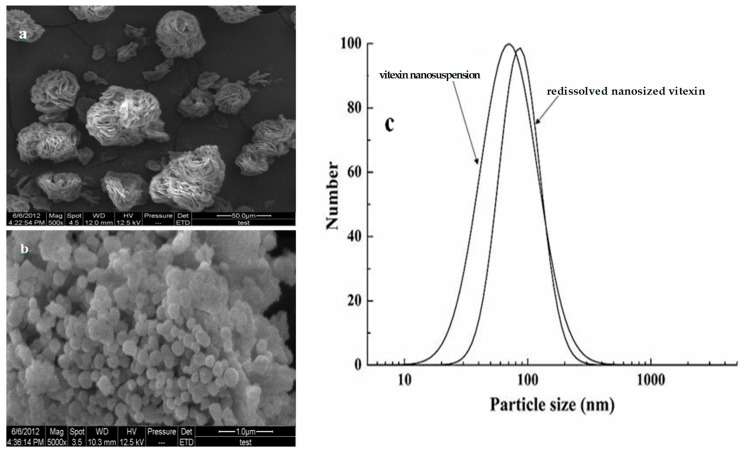
SEM images of (**a**) raw vitexin, (**b**) vitexin nanoparticles, and (**c**) Particle size distribution (PSD) of redissolved vitexin nanoparticles.

SEM images of raw and vitexin nanoparticles were shown in Figure 4. As shown in Figure 4a, the raw vitexin formed rose-like crystals with particle size distributing between 10 and 70 µm. However, in Figure 4b, vitexin nanoparticles were almost spherical with particle size ranging from 150 to 200 nm. Vitexin nanoparticles were significantly smaller and more uniform than raw vitexin, which indicates better solubility. Figure 4c showed the particle size distribution of vitexin nanosuspensions and redissolved nanosized vitexin. The vitexin nanosuspensions with an MPS of 80.5 nm were obtained under optimum preparation conditions. Compared to vitexin nanosuspensions, the MPS of redissolved vitexin nanopartilces (108 nm) increased slightly. The major reason for this phenomenon may be explained by the agglomeration of vitexin particles during the freeze drying. Furthermore, the zeta potential values of the vitexin particles obtained through ASP and vitexin nanosuspensions with poloxamer 188 as a stabilizer by ASP-HPH were determined as −3.2 and −18.5 mV, respectively. Poloxamers 188, a nonionic linear copolymers with surfactant properties, was often used as stabilizing, solubilizing or emulsifying agents in pharmaceutical formulations of poorly water soluble drugs [24]. In this study, as an efficient steric stabilizer, poloxamer 188 increased the physical stability of vitexin nanoparticles.

#### 2.4.2. FTIR Assay and LC-MS Assay

**Figure 5:**
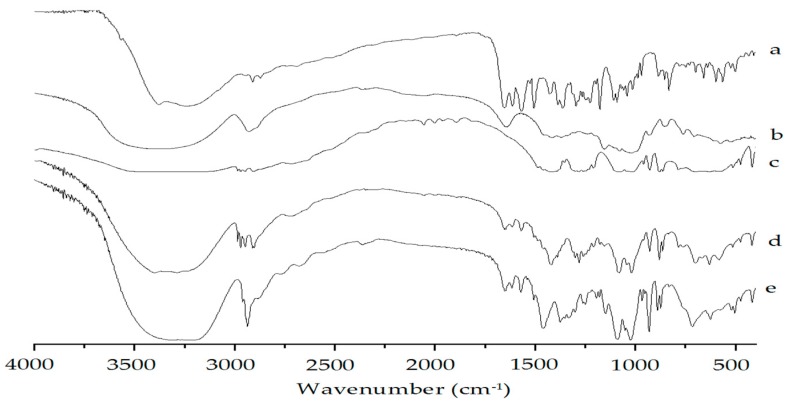
FTIR spectra of (**a**) raw vitexin; (**b**) poloxamer 188; (**c**) mannitol; (**d**) physical mixture of vitexin; poloxamer 188 and mannitol; and (**e**) vitexin nanoparticles.

In FT-IR spectra (Figure 5a), raw vitexin showed characteristic peaks at 3383 (hydroxyl) and 1654 cm^−1^ (carbonyl), as well as at 1614, 1508 and 1429 cm^−1^ (aromatic double bond). The presence of all major peaks of vitexin in physical mixture (Figure 5d) and freeze-dried sample (Figure 5e) ruled out any chemical interaction between vitexin and poloxamer 188. It implied that poloxamer 188 stabilizes vitexin nanoparticles just by physical interaction namely, adsorbing onto the surface of vitexin nanoparticles formed. Furthermore, the chemical structure of vitexin was not changed before and after the formulation and not influenced by excipients in the physical mixture.

**Figure 6:**
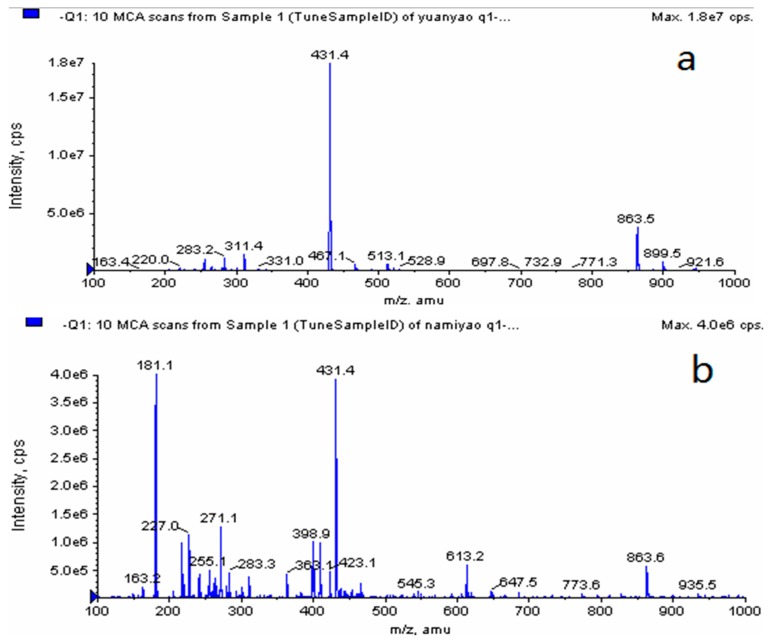
LC-MS spectra of (**a**) raw vitexin and (**b**) vitexin nanoparticles.

The mass spectrograms of raw vitexin and vitexin nanoparticles are shown in Figure 6, in the chart, the molecular weight of unprocessed vitexin and vitexin nanoparticles were both 431.4. Combining the results of FTIR and MS, the molecular structure of nanosized vitexin did not change in the preparation process.

#### 2.4.3. XRD Analysis

**Figure 7:**
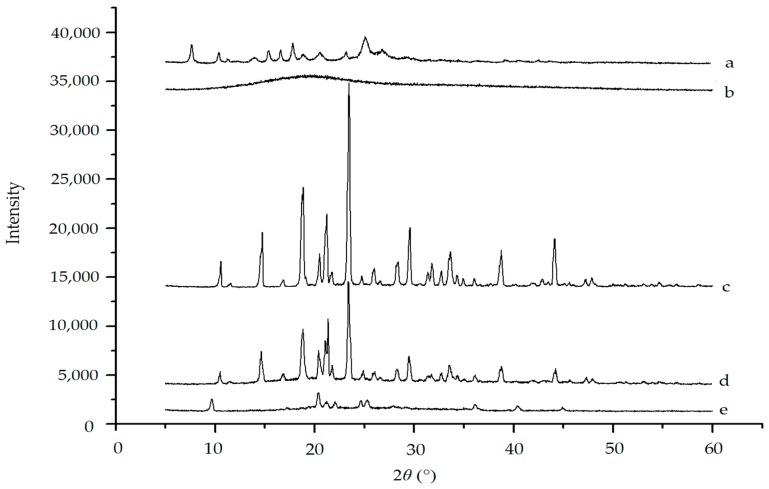
XRPD spectrum of (**a**) raw vitexin; (**b**) poloxamer 188; (**c**) mannitol; (**d**) physical mixture; and (**e**) vitexin nanoparticles.

The X-ray diffraction results for raw vitexin, blank excipients, physical mixture and vitexin nanoparticles are shown in Figure 7. As shown in Figure 7a, diffraction peaks of raw vitexin were observed at the diffraction angles of 2*θ* = 7.66°, 10.40°, 11.24°, 14.12°, 15.36°, 16.6°, 17.8°, 18.72°, 20.56°, 23.20°, 25.04°, 26.80° and 29.10°. In Figure 7d, characteristic diffraction peaks of physical mixture were almost the same as those of mannitol. Diffraction peaks of raw vitexin might be covered or overlapped by those of mannitol. The vibration intensity of the diffraction peaks of the vitexin nanoparticles obviously decreased compared with physical mixture, and the diffraction peaks of mannitol can be observed in physical mixture or vitexin might be present in an amorphous state. Changes in crystallinity of mannitol could occur during the freeze-drying process, which caused its characteristic diffraction peaks to become weak or disappear. Vitexin nanoparticles were found a complete lack of any diffraction peak compared to raw vitexin, which implied that the nanosized vitexin existed in the amorphous state instead of crystalline form. It was reported that the lyophilization process step can modify the structure of drug powder [25,26].

#### 2.4.4. Residual Solvent Determination

**Figure 8:**
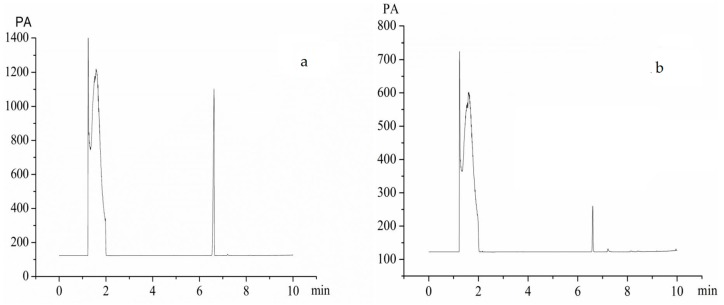
Gas chromatograms of samples (**a**) DMSO standard solution; and (**b**) vitexin processed by the ASP process.

Dimethyl sulfoxide (DMSO), a class 3 solvent with low toxicity in humans, was applied to prepare vitexin particles through the ASP process. Figure 8 showed the GC analysis results of residual DMSO. Seen from Figure 8a, H_2_O and DMSO were eluted at 1.2 and 6.6 min, respectively. A linear regression equation of peak area (*Y*) versus DMSO concentration (*x*) was obtained as *Y* = 0.7235*x* − 25.608 (*R*^2^ = 0.9988) over the range of 0.2–5 mg/mL. Based on the equation, the amount of residual DMSO in vitexin particles is 1757 ppm, which is much lower than the ICH limit (below 5000 ppm) and is acceptable for pharmaceutical use.

#### 2.4.5. Dissolution Rate Analysis

**Figure 9:**
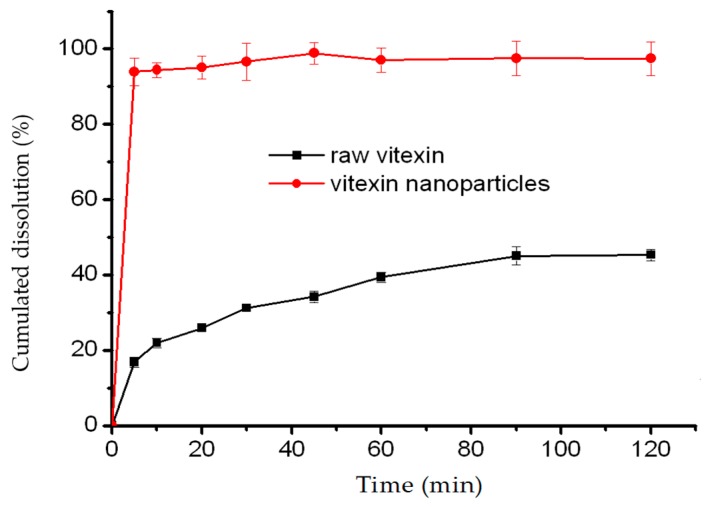
Dissolution profiles of raw vitexin and vitexin nanoparticles.

As seen in Figure 9, obvious differences were observed between the two in dissolution curves. Vitexin nanoparticles showed improved dissolution rate as compared to raw vitexin. In the 5 min, the in vitro dissolution rates of raw vitexin and nanoparticles were around 16.85% and 93.94%, respectively. However, at 30 min, dissolution rates of raw vitexin and nanoparticles were found to be around 31.17% and 96.58%, respectively. The accelerated dissolution rate of vitexin nanoparticles could be mainly explained by the particle size reduction, resulting in a significant increase in specific surface area [27,28].

## 3. Materials and Methods 

### 3.1. Materials

Vitexin (FW = 432.4, purity 99.7%) was provided from Chengdu Biopurify Phytochemicals Ltd. (Chengdu, China). Dimethyl sulfoxide (DMSO, purity ≥ 98.5%) and acetonitrile of chromatographic grade were purchased from Sigma Aldrich (Steinheim, Germany). Poloxamer 188 was purchased from German BASF Company (Ludwigshafen, Germany). Deionized water was purified by a Milli-Q water purification system (Millipore Co., Bedford, MA, USA).

### 3.2. Preparation of Vitexin Nanosuspensions by the ASP-HPH Approach

#### 3.2.1. ASP Process

In preliminary experiments, the solubility of raw vitexin in different organic solvents such as DMSO, ethanol, acetone, isopropanol, n-butanol, ethyl acetate and *N*-methylpyrrolidone was investigated, respectively. It was found that vitexin shows the good solubility only in DMSO. Hence, DMSO was selected as a solvent in the ASP process. The ASP process is conducted as follows: DMSO and deionized water were used as solvent and antisolvent, respectively. A certain amount of raw vitexin was completely dissolved in DMSO at different concentrations. The obtained drug solution was then poured into deionized water with vigorous magnetic stirring. Vitexin particles immediately precipitated from the solution upon mixing. The stirring intensity and temperature were controlled by a temperature-controlled magnetic stirrer. After stirring for 6 min, micro/nanoparticle suspensions were centrifuged at 12,000 rpm for 10 min and washed three times with deionized water.

The factors affecting particle size such as vitexin solution, antisolvent to solvent volume ratio, stirring speed and precipitation temperature were optimized by an orthogonal array design OAD9 (3^4^), which was shown in Table 3. The optimization of the ASP process was carried out with four factors and three levels, namely, concentration of vitexin solution (25, 35, 45 mg/mL), antisolvent to solvent volume ratio (5, 15, 25), stirring speed (500, 1000, 1500 rpm) and precipitation temperature (4, 25, 35 °C). According to the preliminary experimental results, the ranges and levels of influencing factors were confirmed. The MPS (nm) of vitexin particles was the dependent variable. The data were analyzed using the Design-Expert software (Version 8.0.5, Stat-Ease Inc., Minneapolis, MN, USA). Differences at a level of *p* < 0.05 were considered statistically significant.

#### 3.2.2. HPH Process

Subsequently, 50 mg of vitexin particles obtained under the optimal ASP conditions were dissolved in 50 mL distilled water containing 5% (*v*/*w*) Poloxamer 188 as stabilizers to prevent the aggregation of particles in suspensions.

The coarse suspensions were homogenized by a high pressure homogenizer (AH100D, ATS Engineering Inc., Vancouver, BC, Canada) under different operating conditions (homogenization pressure varying from 200 to 1200 bars and cycle numbers ranging from 5 to 25). The effects of homogenization pressure and cycle number on the MPS of vitexin nanosuspensions were optimized by a single factor optimization design. The particle size was determined by particle size analyzer laser (ZetaPALS, Brookhaven Instruments Co., Holtsville, NY, USA).

#### 3.2.3. Lyophilization

The obtained vitexin nanosuspensions were then lyophilized by the addition of 5% (*w*/*v*) mannitol as a cryoprotectant. The nanosuspensions were prefrozen in the refrigerator (DW-86L, Haier, Qingdao, China) at −40 °C for 24 h and then lyophilized using a lyophilizer (Beijing Boyikang Lab Instrument Co., Ltd., Beijing, China) at −50 °C for 48 h. Freeze-dried samples were used for solid state characterization.

### 3.3. Characterization Vitexin Nanoparticles

#### 3.3.1. Morphology, MPS and Zeta Potential Measurement

The surface morphology of raw vitexin and vitexin nanoparticles were observed by SEM (Quanta 200, FEI Company, Eindhoven, The Netherlands). The MPS of vitexin nanosuspensions and the particle size distribution (PSD) of lyophilized powder dispersed in deionized water were measured with dynamic light scattering (DLS) (Zeta-PALS, Brookhaven Instruments Corp., Holtsville, NY, USA). The zeta potential values of vitexin particles obtained through ASP and vitexin nanosuspensions prepared by ASP-HPH techniques were measured using a PALS Zeta Potential Analyzer (Brookhaven Instruments Corp., Holtsville, NY, USA) with the samples diluted in deionized water to approximately 1 mg/mL for each measurement. All measurements were made in triplicate.

#### 3.3.2. FTIR Analysis

Chemical bond status of the samples was analyzed with FTIR (IRAFFINITY−1, Shimadzu Co. Kyoto, Japan.) spectra using KBr in the wave number range of 4000–500 cm^−1^ at a resolution of 4 cm^−1^.

#### 3.3.3. LC-MS Analysis

The raw vitexin and vitexin nanoparticles were dissolved separately in HPLC grade methanol. An Agilent 1100 series HPLC system (Agilent Inc., Palo Alto, CA, USA) consisted of a G1312A binary pump, a 7725i manual injector, and a G1379A degasser. The analytical column employed was a HiQ sil C18W (4.6 mm φ × 250 mm, 5 μm). The mobile phase was methanol–water–formic acid (15:84.99:0.01, *v*/*v*/*v*). The flow rate was 1 mL/min and the column temperature was 25 °C. The column effluent was monitored by an API3000 triple quadrupole mass spectrometer (Biosystems, Toronto, ON, Canada) with an electrospray ionization (ESI) source, which was connected to the liquid chromatography system. Ionization of vitexin was obtained in the negative ion mode. Compound parameters, viz. declustering potential (DP) and entrance potential (EP) were set at −70 and −10 V, respectively, for analysis of vitexin. The Analyst 1.4 of API3000 (AB, Foster City, CA, USA) was used for data acquisition.

#### 3.3.4. XRPD Analysis

XRPD analysis of different samples was measured by X-ray diffraction (JDX-3530, JEOL, Tokyo, Japan). The pattern was scanned in the range of diffraction angle 2*θ* = 5–60°at a rate of 0.02°/min with a step length of 2*θ* = 0.02° using Cu Kα1 radiation at 40 kV and 40 mA.

#### 3.3.5. Residual DMSO Determination

GC analysis of the residual DMSO in ASP process was determined by an Agilent 6890N gas chromatograph (Agilent Technologies, Palo Alto, CA, USA) equipped with a flame ionization detector (FID) detector and a HP-5 capillary column (30 m × 0.32 mm × 0.25 μm, 5% phenyl methyl siloxane). In addition, 2.0 g of vitexin particles was dissolved in 10 mL deionized water. The vitexin solution was shaken by ultrasonic irradiation at 25 °C for 60 min. The sample was then centrifuged at 15,000 rpm for 15 min, and 5.0 μL of resulting supernatants was injected into the GC injection port. The GC settings were programmed as follows: the initial oven temperature was maintained at 100 °C for 3 min, and ramped at 2 °C/min to 200 °C. Nitrogen was employed as a carrier gas with a flow rate of 1 mL/min. Both the inlet and the detector temperatures were kept at 250 °C and the GC was run in a split mode set at a 1:1. Each determination was done in triplicate.

#### 3.3.6. Dissolution Test 

The dissolution study of vitexin nanoparticles, equivalent to 20 mg of raw vitexin, was performed by the paddle method. In addition, 200 mL of phosphate buffer solution pH 7.4 at 37.0 ± 0.5 °C was adopted as a dissolution medium at a paddle speed of 100 rpm. The raw drug and nanoparticles were dispersed in 200 mL dissolution medium accordingly. Furthermore, 1 mL of samples was withdrawn from the dissolution vessel at 5, 10, 20, 30, 45, 60, 90 and 120 min, filtered through a 0.22 μm filters and analyzed by the previously described HPLC method. Each sample was done in triplicate. The cumulative release amount of drug versus time profiles was plotted.

## 4. Conclusions

In summary, we prepared the nanoparticles of vitexin by combining the antisolvent precipitation and high pressure homogenization approaches followed by lyophilization. The vitexin nanosuspensions with an MPS of 80.5 nm were firstly produced under the following optimum ASP and HPH operating conditions (i.e., vitexin solution concentration 25 mg/mL, antisolvent to solvent volume ratio 15 times, stirring speed 1500 rpm, precipitation temperature 4 °C, homogenization pressure 800 bar and number of homogenization cycles 20) and then lyophilized to form nanoparticles. The obtained nanoparticles of vitexin existed in an amorphous form, with its chemical structure unchanged. Additionally, the DMSO residual is much lower than the ICH limit for class 3 solvents. The dissolution rate of vitexin nanoparticles was greatly improved compared to the raw drug. Overall, the combinative process we developed is an effective way to produce vitexin nanoparticles with a markedly enhanced dissolution rate.

## Figures and Tables

**Table 1:** OAD9(3^4^) and experimental results.

Trial No.	A	B	C	D	MPS (nm) ± SD (*n* = 3)
1	1(25)	1(5)	1 (500)	1(4)	425.8 ± 45.22
2	1(25)	2(15)	2(1000)	2(25)	404.4 ± 34.83
3	1(25)	3(25)	3(1500)	3(35)	775.8 ± 29.12
4	2(35)	1(5)	2(1000)	3(35)	1129.2 ± 95.25
5	2(35)	2(15)	3(1500)	1(4)	368.4 ± 31.23
6	2(35)	3(25)	1(500)	2(25)	817.6 ± 70.69
7	3(45)	1(5)	3(1500)	2(25)	880.9 ± 50.18
8	3(45)	2(15)	1(500)	3(35)	1073.6 ± 88.90
9	3(45)	3(25)	2(1000)	1(4)	459.0 ± 28.92
*K*_1_^(a)^	535.33 ± 36.39	901.967 ± 65.81	772.333 ± 68.27	417.733 ± 35.12	
*K*_2_	861.73 ± 67.98	615.467 ± 51.65	754.2 ± 55.26	700.967 ± 51.90	
*K*_3_	804.50 ± 56.00	684.133 ± 42.91	675.033 ± 36.84	1082.867 ± 73.35	
*R* ^(b)^	326.40	286.50	97.30	665.13	
Optimal level	A1	B2	C2	D1	

A: concentration of vitexin solution (mg/mL); B: antisolvent to solvent volume ratio; C: stirring speed (rpm); D: reaction temperature. ^(a)^ *K_i_*^A^ = Σ (mean particle size at *A_i_*)/3, the mean values of the mean particle size for a certain factor at each level with standard deviation. ^(b)^ *R_i_*^A^ = max{*K_i_*^A^} − min{*K_i_*^A^}.

**Table 2:** ANOVA analysis of four factors for the ASP process.

Factor	Sum of Squares	Degrees of Freedom	*F* Ratio	*F*_0.05_	Type of Effect
A: Concentration of vitexin solution (mg/mL)	182,263.31	2	11.35	19.00	
B: Antisolvent to solvent volume ratio	134,248.72	2	8.36	19.00	
C: Stirring speed (rpm)	16,063.47	2	1.00	19.00	
D: Reaction temperature	668,471.08	2	41.61	19.00	significant
Error	16,063.47	2			

**Table 3:** Factors and levels of the OAD.

Factor/Level	A	B	C	D
	Concentration of Vitexin Solution (mg/mL)	Antisolvent to Solvent Volume Ratio	Stirring Speed (rpm)	Precipitation Temperature (°C)
1	25	5	500	4
2	35	15	1000	25
3	45	25	1500	35

## Abbreviations

ASP	antisolvent precipitation
HPH	high pressure homogenization
OAD	orthogonal array design
MPS	mean particle size
ICH	International Conference on Harmonization
DMSO	Dimethyl sulfoxide
SEM	Scanning electron microscopy
FTIR	Fourier transform infrared spectroscopy
LC-MS	Liquid chromatography–mass spectrometry
XRPD	X-ray powder diffraction
GC	Gas chromatography
